# Prediction of soil organic carbon in a coal mining area by Vis-NIR spectroscopy

**DOI:** 10.1371/journal.pone.0196198

**Published:** 2018-04-20

**Authors:** Wenjuan Sun, Xinju Li, Beibei Niu

**Affiliations:** College of Resources and Environment, Shandong Agricultural University, Taian, China; RMIT University, AUSTRALIA

## Abstract

Coal mining has led to increasingly serious land subsidence, and the reclamation of the subsided land has become a hot topic of concern for governments and scholars. Soil quality of reclaimed land is the key indicator to the evaluation of the reclamation effect; hence, rapid monitoring and evaluation of reclaimed land is of great significance. Visible-near infrared (Vis-NIR) spectroscopy has been shown to be a rapid, timely and efficient tool for the prediction of soil organic carbon (SOC). In this study, 104 soil samples were collected from the Baodian mining area of Shandong province. Vis-NIR reflectance spectra and soil organic carbon content were then measured under laboratory conditions. The spectral data were first denoised using the Savitzky-Golay (SG) convolution smoothing method or the multiple scattering correction (MSC) method, after which the spectral reflectance (R) was subjected to reciprocal, reciprocal logarithm and differential transformations to improve spectral sensitivity. Finally, regression models for estimating the SOC content by the spectral data were constructed using partial least squares regression (PLSR). The results showed that: (1) The SOC content in the mining area was generally low (at the below-average level) and exhibited great variability. (2) The spectral reflectance increased with the decrease of soil organic carbon content. In addition, the sensitivity of the spectrum to the change in SOC content, especially that in the near-infrared band of the original reflectance, decreased when the SOC content was low. (3) The modeling results performed best when the spectral reflectance was preprocessed by Savitzky-Golay (SG) smoothing coupled with multiple scattering correction (MSC) and first-order differential transformation (modeling R^2^ = 0.86, RMSE = 2.00 g/kg, verification R^2^ = 0.78, RMSE = 1.81 g/kg, and RPD = 2.69). In addition, the first-order differential of R combined with SG, MSC with R, SG together with MSC and R also produced better modeling results than other pretreatment combinations. Vis-NIR modeling with specific spectral preprocessing methods could predict SOC content effectively.

## Introduction

Traditional methods for the determination of soil organic carbon (SOC) content not only are time consuming and laborious but also need high cost and exhibit poor real-time performance [1]. Hyperspectral data, via narrow and fine spectral bands, can capture the deep information hidden in the soil. And it was widely used in analysis of soil physical and chemical properties, such as soil humus structure [2], soil nutrients [3–4], soil salinity [5] and soil moisture content [6]. All above studies have confirmed that hyperspectral data had the advantage of real time, high efficiency and low cost, which could make up the shortcomings of the traditional methods primely.

In the field of SOC research, Vis-NIR spectroscopy is mainly used for spectral feature analysis and quantitative prediction. Bartholomeus [7] analyzed nine kinds of soil samples with SOC contents varying from 0.06% to 45.1% and concluded that the spectral index (which was defined as the sum of the total reflectance minus the continuum removed function) based on visible light correlated well with SOC. Henderson [8] found that there was good correlation between organic carbon in soils derived from different parent materials and near-infrared longwave bands (1100~2526 nm). Consorti suggested that laboratory reflectance spectroscopy in the Vis-NIR range coupled with a geostatistical analysis can be used as a tool for predicting spectrally mapped SOM [9]. Aïchi et al. [10] established a model that was proven to be valid over a range of 0.90–5.20% of organic carbon content through the original spectral absorbency of 400~950 nm, which included the near-infrared band. The above studies have shown that visible and near-infrared bands exhibited good statistical relationships with SOC content. Mouazen concluded that appropriate preprocessing of Vis-NIR diffuse reflectance spectra could aid extracting sensitive variables for PLSR modeling to achieve higher prediction accuracy of soil total N, total C, and organic C [11]. However, few study has been done to improve the prediction accuracy using a combination of various spectral preprocessing methods.

Although the exploitation of coal resources has promoted economic development, it has also caused severe surface collapse problems, directly leading to deterioration of soil quality [12]. While land reclamation can improve soil structure and soil physical and chemical properties, thereby increasing soil quality [13]. Although SOC accounts for only a small proportion of total soil, it plays a key role in regulating soil quality and function. SOC is not only a carbon source for the growth of microorganisms, vegetation and saprophytic animals, but also an important ecological factor that can maintain sound soil physical structure and biological diversity [14]. Therefore, the rapid monitoring and evaluation of the SOC content is important with respect to reclamation modes and management measures in mining areas [15]. In this study, soil samples were collected in a mining area for the determination of SOC content and Vis-NIR data, and the spectral data were pretreated by different combinations of Savitzky-Golay (SG), multiple scattering correction (MSC) and various mathematical transformations. The transformed data were then modeled and optimized by PLSR to estimate the SOC content in the study area, which can provide a basis for monitoring and evaluation of SOC.

## Methods and materials

### Research area

**Fig 1:**
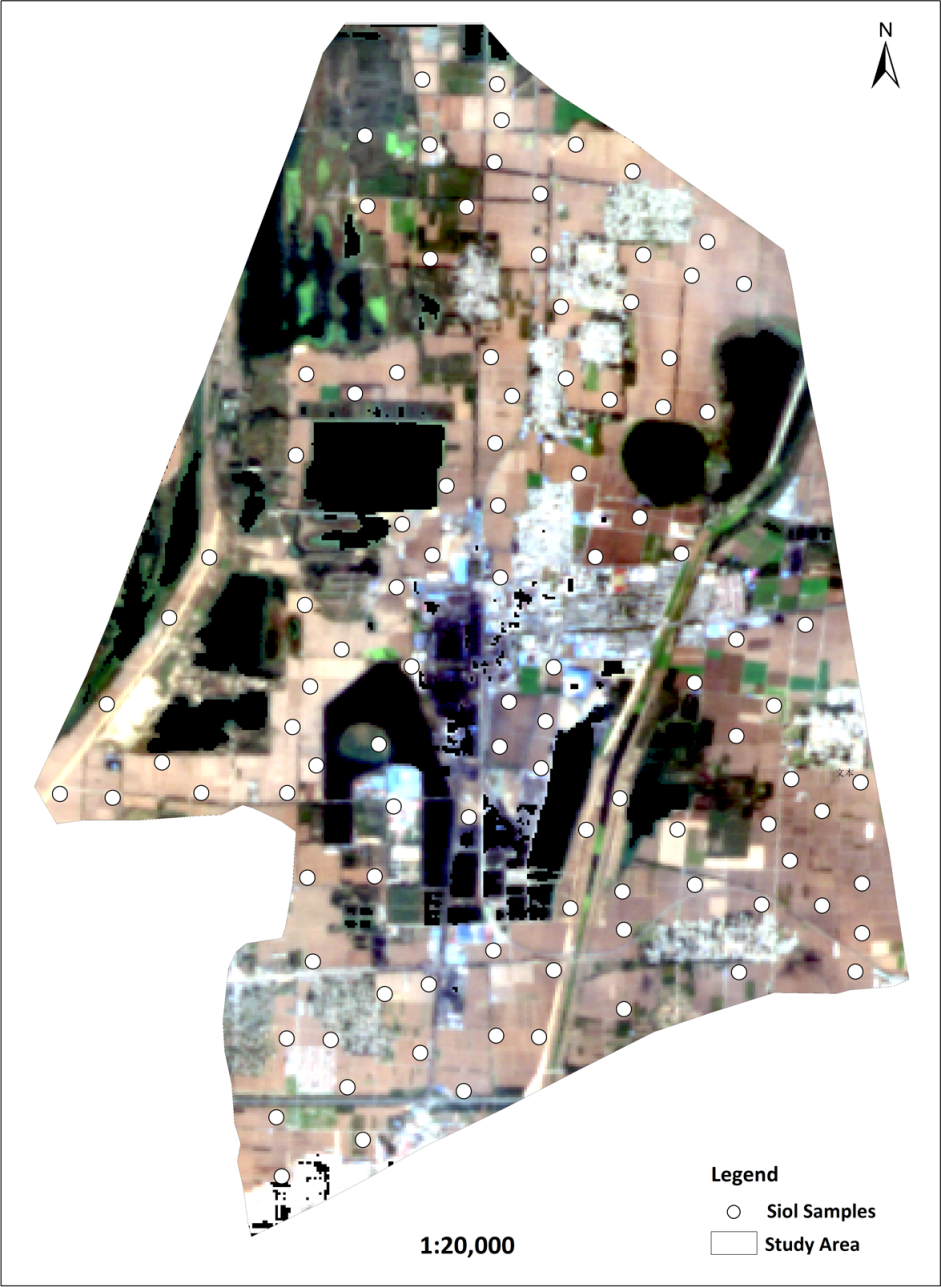
Study area and sample distribution.

The Baodian mining area is located in the northwest region of Zoucheng City, which encompasses a total area of approximately 35.76 km^2^ and lies between 35°23’13.2”-35°28’8.4”N latitude and 116°48’7.2‘‘-116°52’26.4”E longitude. The study area has a temperate continental monsoon climate with four distinct seasons. The topography is dominated by plains, and the soil types are mainly fluvo-alluvial soil and Vertisol. Since 1986, long-term coal mining activities have caused serious ground subsidence problems, and the current water-logged area occupies nearly 1/3 of the area (Fig 1).

### Sampling and analysis of soil samples

The sampling work was carried out during 2–7 June 2017 after wheat crops were harvested. The sampling sites were arranged as evenly as possible according to actual situation in the study area, and a total of 104 soil samples were collected. The position of each sampling site was determined using GPS, and approximately 1.0 kg surface soil (0–20 cm) was collected at each site. A mixture of 5 pieces of soil was collected using a diagonal sampling method within an area of 10 m×10 m. Each soil sample was kept in a sealed package for subsequent spectral measurement and SOC content determination in the laboratory.

The plant roots, stones, small animals and other intrusions mixed in the soil samples were first removed, and then dried and ground until the particles could pass through a 20-mesh sieve and then a 100-mesh sieve (the 20-mesh sieve was used for later spectral measurements). The soil samples that passed through the 100-mesh sieves were used for the determination of the SOC content via potassium dichromate titration method.

### Determination and processing of soil spectra

The spectral determination was carried out in a darkroom using an ASD FieldSpec4 Spectroradiometer, whose spectral range is 350–2500 nm. In spectral measurements, the probe field angle was adjusted to 15°, and the incident angle of the light source was 30°; the distances from the light source and probe to the soil surface were 50 cm and 15 cm, respectively. The soil samples were sieved through 20-mesh screen for spectral measurements. To avoid the influence of ground-reflected light, the soil was loaded into an aluminum case, under which a light-absorbing cloth was placed. And the surface of the aluminum case was scraped with cardboard for a smooth surface of the soil [16]. 8 spectral curves for each soil sample were detected and collected.

The mean spectrum of the 8 curves measured for each sample was used as the measured sample curve. The data were then exported to EXCEL, except the data of the initial band (350–499 nm), the tail band (2451–2500 nm), and the bands influenced by water vapor in the environment (1300–1450 nm and 1800–1950 nm). Then, the spectral data were processed in three different ways (SG, MSC, and SG together with MSC). Then the preprocessed reflectivity was subjected to reciprocal (1/R), reciprocal logarithm (log(1/R)), first-order differential (R’) and second-order differential (R”) transformations. A total of 15 kinds of modeling data were generated. Previous studies have shown that spectral noise can be effectively reduced when the SG smoothing data window is approximately 15, the fitting order is 3, and 2 smooth times were used [17]. MSC can also effectively eliminate the scattering effects caused by particle size, loading density and humidity [18]. Mathematical transformation can effectively extract soil spectral characteristics and prominent hidden spectral information [19].

### Partial least squares regression analysis

In this study, R and its transformed form were independent variables. The bands of hyperspectral were narrow and multiple, and they were closely correlated with the adjacent bands. Therefore, on the one hand, the number of independent variables was much larger than the sample number; on the other hand, there was a high degree of autocorrelation within the independent variables. PLSR, combined with the principal component analysis, canonical correlation analysis and OLS regression [20], can effectively solve the abovementioned problems.

Modeling was performed by R software. The coefficient of determination (R^2^), root mean square error (RMSE) and the relative prediction deviation (RPD) were used to evaluate the accuracy of the models. A higher R^2^ indicates a better degree of model fitting, and a lower RMSE indicates more accurate model prediction. Models with a higher RPD (greater than 2) are more robust. In summary, the models with higher R^2^ and RPD values but lower RMSE values are much more reliable.

## Results and analysis

### Statistical characteristics of organic carbon in soil samples

The total soil samples were divided into a training set (64 samples) and a verification set (40 samples) based on the spectral reflection characteristics by the K-S algorithm.

In terms of the statistical characteristics of the sample collectivity, the SOC content ranged from 0.79 g/kg to 27.72 g/kg, with an average value of 11.34 g/kg, indicating that the SOC content in the study area was generally low (at the below-average level). The standard deviation and coefficient of variation were 5.09 g/kg and 44.86%, respectively, indicating that the sample has a certain discreteness. The parameter values in the training set and verification set were similar to those in the sample collectivity (Table 1).

**Table 1:** Statistical parameters of organic carbon in soil samples.

Soil sample set	Minimum value (g•kg^-1^)	Maximum value (g•kg^-1^)	Mean value (g•kg^-1^)	Standard deviation (g•kg^-1^)	Variation coefficient (%)
All samples	0.79	27.72	11.34	5.09	44.86
Training set	1.19	27.72	11.55	5.14	44.50
Validation set	0.79	27.33	10.97	5.01	45.88

### Spectral characteristics of soil organic carbon

In accordance with the classification standard of soil organic matter in the second national soil survey in China [21], the soil samples were divided into 6 groups (grade I, >23.20 g/kg; grade II, 17.40–23.20 g/kg; grade III, 11.60–17.40 g/kg; grade IV, 5.80–11.60 g/kg; grade V, 3.48–5.80 g/kg, and grade VI, 0.58–3.48 g/kg) based on their SOC content. The mean soil spectrum of each group was used to analyze the spectral characteristics of the SOC content at different grades.

**Fig 2:**
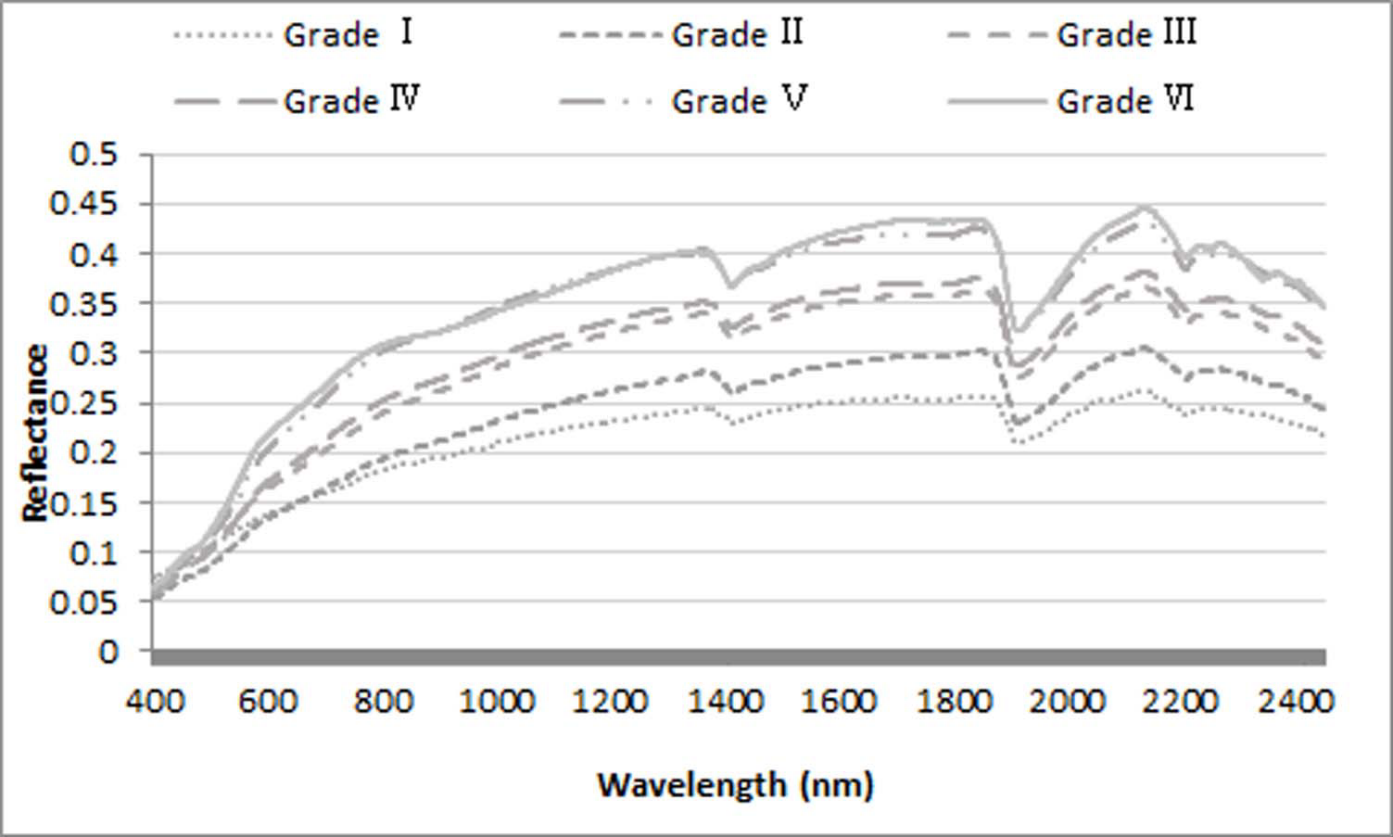
Spectral characteristics of soils with different SOC contents.

Spectral characteristics of soils with different SOC grades were demonstrated in Fig 2, and it can be seen that R was negatively related to the SOC content, which was obvious within grades I, II, and III, while R varied slightly between grades V and VI. The spectral curves of the soil with different SOC content grades exhibited a uniform pattern, which increased rapidly in the visible band (400–760 nm) but then ascended gradually in the short near-infrared and near-infrared longwave bands (780–1300 nm), then the curves formed a high reflectivity platform until it began to decline after 2100 nm. As ambient water absorption electromagnetic waves are strong near 1400 nm and 1900 nm, two absorption valleys were formed. There was a reflection peak near 2150 nm that reaches the maximum reflectivity.

### Establishment and optimization of hyperspectral prediction models for soil organic carbon

As shown in Table 2, a total of 15 sets of modeling data were used to construct the prediction model for SOC content by PLSR. The modeling results from log(1/R) and R” were both inferior to those from R, while R’ can improve the model accuracy indeed. The spectral data processed by MSC produced better results in F(R) (the inversion model that took R as the independent variable). Among models with SG preprocessing, F(R’) (the inversion model that took R’ as the independent variable) had a higher modeling accuracy; and among models with SG+MSC preprocessing, both F(R) and F(R’) were better than the others. Overall, the preferable models were F(R’) with SG preprocessing, F(R) with MSC preprocessing, and F(R’) or F(R) with SG+MSC preprocessing.

**Table 2:** Partial least squares modeling results of soil organic carbon.

Model	Parameters		SG	MSC	SG+MSC
F(R)^a^	Training set	R^2^	0.64	0.84	0.8
		RMSE	3.21	2.11	2.36
	validation set	R^2^	0.35	0.72	0.71
		RMSE (g•kg^-1^)	3.53	2.04	2.27
		RPD	1.47	2.25	2.24
F(1/R)^a^	Training set	R^2^	0.55	0.44	0.75
		RMSE (g•kg^-1^)	2.7	4.37	2.97
	validation set	R^2^	0.28	0.18	0.39
		RMSE (g•kg^-1^)	4.23	4.01	4.75
		RPD	1.12	1.12	1.17
F(lg(1/R))^a^	Training set	R^2^	0.52	0.23	0.46
		RMSE (g•kg^-1^)	2.87	4.93	4.16
	validation set	R^2^	0.36	0.12	0.21
		RMSE (g•kg^-1^)	3.78	4.17	3.68
		RPD	1.24	1.03	1.09
F(R’)^a^	Training set	R^2^	0.71	0.68	0.86
		RMSE (g•kg^-1^)	2.92	2.3	2
	validation set	R^2^	0.66	0.24	0.78
		RMSE (g•kg^-1^)	1.91	5.64	1.81
		RPD	2.44	1.01	2.69
F(R”)^a^	Training set	R^2^	0.28	0.15	0.25
		RMSE (g•kg^-1^)	4.23	4.98	4.66
	validation set	R^2^	0.19	0.01	0.12
		RMSE (g•kg^-1^)	5.13	5.94	5.74
		RPD	1.01	0.9	0.98

^a^ F(x) is a model with “x” as an independent variable, X = R, 1/R, lg(1/R), R’, R”.

**Fig 3:**
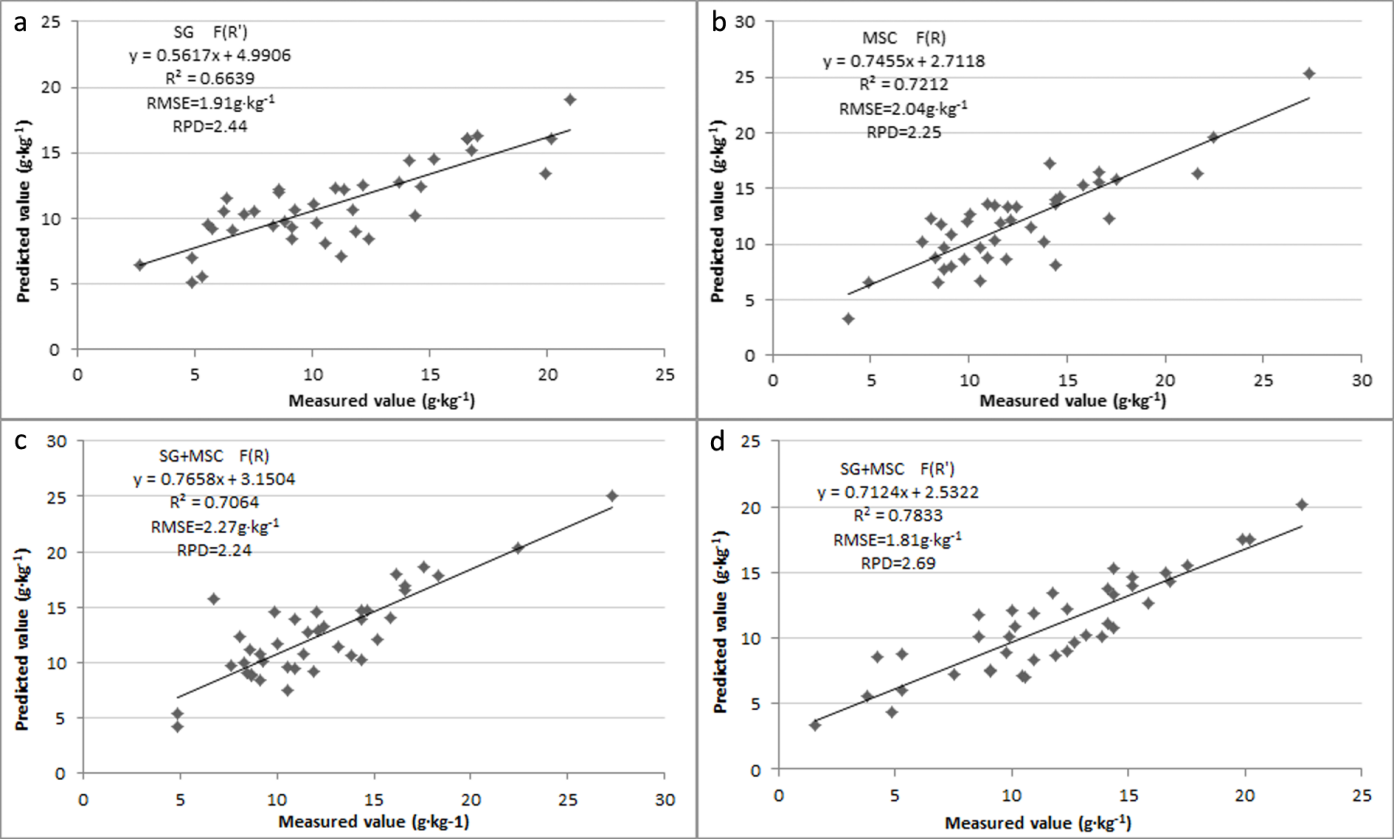
Relationship between measured and predicted soil organic carbon.

The predicted and observed values of the model were verified and analyzed. In terms of the results (Fig 3), the following four models exhibited good fitting, especially the models whose R were preprocessed using SG, MSC, and first-order differential transformations (Fig 3D); these models could effectively predict the SOC content.

## Discussion

This study demonstrated that the SOC content was negatively correlated with spectral reflectance, which is consistent with many other research findings [22–23]. Preprocessing R with MSC or “SG+MSC” could aid improving the fitting R^2^ (which could reach 0.84) of the constructed estimation models. The R^2^ of F(R’) with SG or “SG+MSC”preprocessing was relatively higher (0.71 and 0.86, respectively) than that of other models, and similar conclusions were also drawn by Wang HT [24] in his study on forest SOC. Fig 3 shows that the R^2^ of F(R) was lower with SG preprocessing and higher with MSC preprocessing. However, the situation was reversed with F(R’). Therefore, it was deduced that preprocessing with SG and R’ transformation can better extract spectral characteristics, and thus get better modeling results (which is consistent with the results of Aixia Yang [25]); By contrast, MSC processing is more suitable for direct modeling using the original reflectivity, and the model accuracy is better than that of F(R’) with SG preprocessing. While this result differed with the study by Huazhou Chen [26], which may be due to differences in the regional environment and soil type [27]).

**Fig 4:**
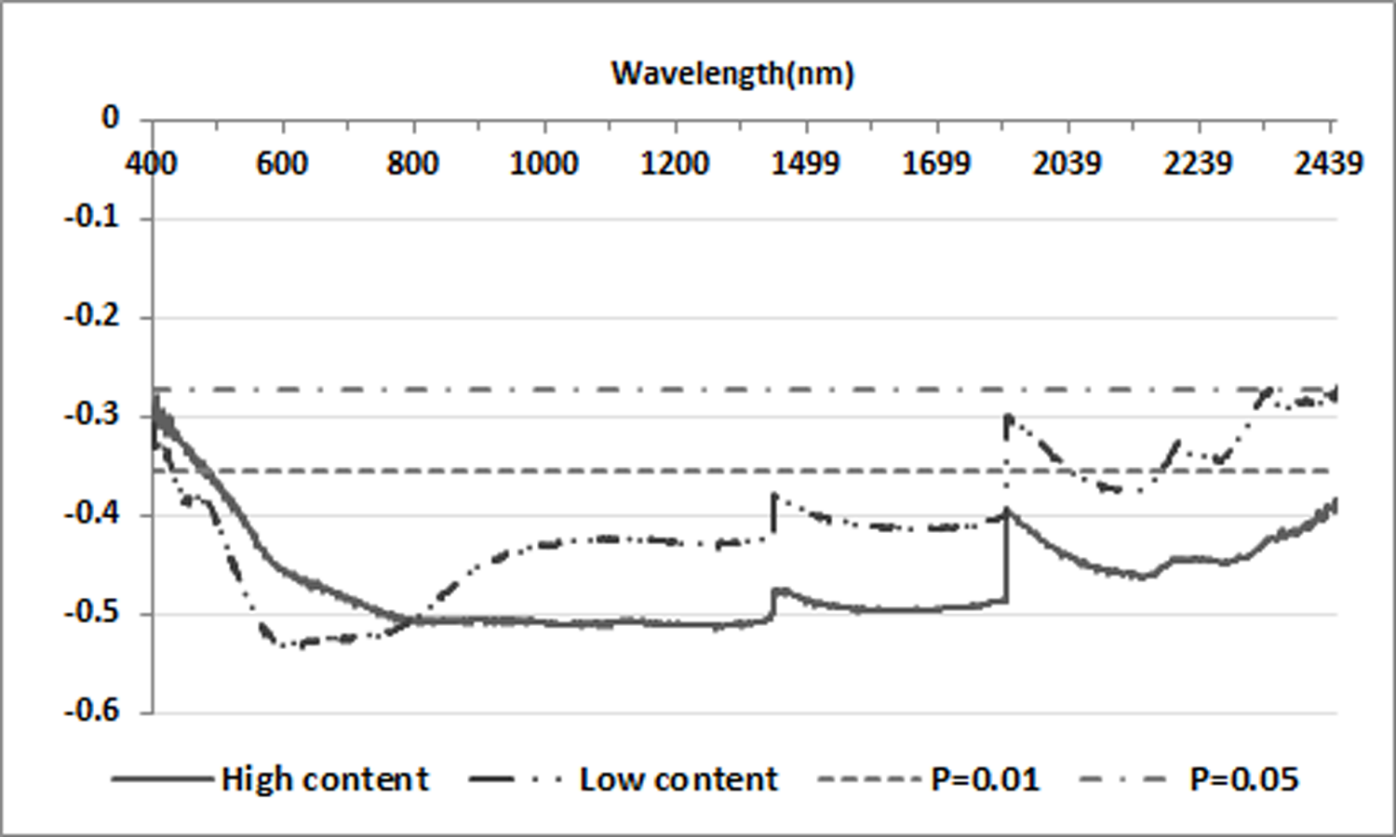
Correlation analysis between different soil organic carbon contents and spectral reflectance.

This study found that when the SOC content decreased to a certain extent, the negative correlation trend between spectral reflectance and SOC content was no longer obvious. Presumably, when the SOC content is low, the spectral characteristic information of SOC may be obscured by other information in the soil spectrum. To further verify this speculation, correlation analysis between different soil organic carbon contents and corresponding spectral reflectances was carried out in two groups divided according to SOC content. As shown in Fig 4, in the range of 400–700 nm, the SOC-reflectance correlation of the low-content group was slightly higher than that of the high content group; in the range of 750–2450 nm (excluding the ranges of 1300–1450 nm, 1800–1950 nm, and 2450–2500 nm), the correlation of the high content group was much higher than that of the low content group. This indicates that the speculation is of certain reliability. When the SOC content is low, it is not suitable for direct modeling because of the low sensitivity of R. However, how low the SOC content is when the hyperspectral model is no longer applicable, still needs further study.

In addition, the SOC content in the study area ranged from 0.79 g/kg to 27.72 g/kg, with an average value of 11.34 g/kg, indicating that the SOC content in the mining area was generally low (at the below-average level) and had great variability. It is speculated that the surface subsidence caused by coal mining would disturb the distribution of SOC and lead to the decrease and regional differences of SOC content, which was also proved by a previous study [12].

## Conclusion

(1) The SOC content in the study area was generally low (at the below-average level) and had great variability.
(2) The spectral curves of soils with different SOC contents were consistent in morphological characteristics, but they were negatively correlated with SOC contents in terms of reflectance values. In addition, the sensitivity of the original reflectance to the change in SOC content decreased when the SOC content was low.
(3) The quantitative prediction of the SOC content in the study area could be effectively determined by PLRS. The extent of model fitting was best up to 0.86, and the verification coefficient was 0.78, and the relative prediction deviation was 2.69.
(4) The modeling results with different spectral processing methods were different. There were four models (F(R’) with SG preprocessing, F(R) with MSC preprocessing, and F(R’) or F(R) with SG+MSC preprocessing) that had relatively better precision, especially the models F(R’) with SG+MSC preprocessing.

## Supporting information

S1 File The primary data of soil organic carbon content and soil spectral reflectance. (XLS)

## References

[1] Srivastava R, Sarkar D, Mukhopadhayay SS, Sood A, Singh M, Nasre RA. Development of hyperspectral model for rapid monitoring of soil organic carbon under precision farming in the Indo-Gangetic Plains of Punjab, India. Journal of the Indian Society of Remote Sensing, 2015; 43(4): 751–759.

[2] Fajardo M, McBratney A, Whelan B. Fuzzy clustering of Vis-NIR spectra for the objective recognition of soil morphological horizons in soil profiles. Geoderma. 2015; 263: 244–253. doi: 10.1016/j.geoderma.2015.05.0105

[3] Wang SQ, Shu N, Zhang HT. In-site total n content prediction of soil with Vis-NIR Spectroscopy. Spectroscopy and Sectral Analysis. 2008; 28(4): 808–812. 18619304

[4] Leone AP, Leone N, Rompone S. An applicantion of VIS-NIR reflectance spectroscopy and artificial neural neworks to the prediction of soil organic carbon content in southern Italy. Fresenius Environmental Bulletin. 2013; 22(4B): 1230–1238.

[5] Liu Y, Pan XZ, Wang CK, Li YL, Shi RJ. Predicting soil salinity with Vis-NIR spectra after removing the effects of soil moisture using external parameter orthogonalization. Plos One. 2015; 10 (10): e0140688 doi: 10.1371/journal.pone.0140688 26468645 10.1371/journal.pone.0140688 PMC4607364

[6] Morellos A, Pantazi XE, Moshou D, Alexandridis T, Whetton R, Tziotzios G, et al Machine learning based prediction of soil total nitrogen, organic carbon and moisture content by using VIS-NIR spectroscopy. Biosystems Engineering. 2016; 152: 104–116. doi: 10.1016/j.biosystemseng.2016.04.018

[7] Bartholomeus HM, Schaepman ME, Kooistra L, Stevens A, Hoogmoed WB, Spaargaren OSP. Spectral reflectance based indices for soil organic carbon quantification. Geoderma. 2008; 145(1): 28–36. doi: 10.1016/j.geoderma.2008.01.010

[8] Henderson TL, Baumgardner MF, Franzmeier DP, Stott DE, Coster DC. High dimensional reflectance analysis of soil organic matter. Soil Science Society of America Journal. 1992; 56(3): 865–872. doi: 10.2136/sssaj1992.03615995005600030031x

[9] Conforti M, Buttafuoco G, Leone A P, et al Studying the relationship between water-induced soil erosion and soil organic matter using Vis–NIR spectroscopy and geomorphological analysis: A case study in southern Italy. Catena, 2013; 110(2): 44–58.

[10] Aïchi H, Fouad Y, Walter C, Rossel RAV, Chabaane ZL, Sanaa M.Regional predictions of soil organic carbon content from spectral reflectance measurements. Biosystems Engineering. 2009;104(3):442–446. doi: 10.1016/j.biosystemseng.2009.08.002

[11] Mouazen A M. Development of Parsimonious Models for Soil N and C Prediction Using Spectral Pretreatments and PLS Regression for VIS-NIR Diffuse Reflectance Spectra. Advances in Biomedical Engineering—Proceedings of 2011 International Conference on Agricultural and Biosystems Engineering. 2011. Available from: http://kreader.cnki.net/Kreader/CatalogViewPage.aspx?dbCode=IPFD&filename=ZNXX201102001041&tablename=IPFD9914&compose=&first=1&uid=WEEvREcwSlJHSldRa1Fhb09jMjVzYjkzQkpibkNJa2lraWsrL0t5Zi9jaz0=$9A4hF_YAuvQ5obgVAqNKPCYcEjKensW4ggI8Fm4gTkoUKaID8j8gFw

[12] Yang D, Bian Z, Lei S. Impact on soil physical qualities by the subsidence of coal mining: a case study in Western China. Environmental Earth Sciences, 2016; 75(8): 1–14.

[13] Dongmei HR. Long Term Effect of Land Reclamation from Lake on Chemical Composition of Soil Organic Matter and Its Mineralization. Plos One. 2014; 9(6): e99251 doi: 10.1371/journal.pone.0099251 24905998 10.1371/journal.pone.0099251 PMC4048317

[14] Wang X, Duan JJ. The status and prospect of soil organic carbon in Southwest China. Tillage and Cultivation. 2016; (5): 80–82.

[15] Bodlák L, Křováková K, Kobesová M, Brom J, St'astny J, Pecharova E. SOC content—An appropriate tool for evaluating the soil quality in a reclaimed post-mining landscape. Ecological Engineering, 2012; 43: 53–59. doi: 10.1016/j.ecoleng.2011.07.013

[16] Hou YP, Lv CW, Xiang HL, Wang H. Treatment effects on soil hyperspectral stability in laboratory test. Chinese Journal of Soil Science. 2015; 46(2): 287–291.

[17] Li PS, Hu S, Sun LS, Sun XX. Coal DTG curve denoise based on Savitzky-Golay method. Journal of Huazhong University of Science and Technology(Nature Science). 2005; 33(7): 61–64.

[18] Guo DD, Huang SM, Zhang SQ, Nie SW. Comparative analysis of various hyperspectral prediction models of fluvo-aquic soil organic matter. Transactions of the Chinese Society of Agricultural Engineering. 2014; 30(21): 192–200.

[19] Tan K, Ye YY, Du PJ, Zhang QQ. Estimation of Heavy Metal Concentrations in Reclaimed Mining Soils Using Reflectance Spectroscopy. Spectroscopy and Spectral Analysis. 2014; (12): 3317–3322. 25881431

[20] Febrero-Bande M, Galeano P. Gonzalez-Manteiga W, Functional principal component regression and functional Partial Least-squares regression: An overview and a comparative study. International Statistical Review. 2017;85(1): 61–83. doi: 10.1111/insr.12116

[21] China Soil Census Office. Chinese soil. Beijing: China Agricultural Press; 1998.

[22] Gao L, Chen YY, Shi TZ, Zhao C, Liu YL, et al Exploring the role of the spatial characteristics of visible and near-Infrared reflectance in predicting soil organic carbon density. ISPRS International Journal of Geo-Information. 2017; 6(10): 308 doi: 10.3390/ijgi6100308

[23] Bao NS, Wu LX, Ye BY, Yang K, Zhou W. Assessing soil organic matter of reclaimed soil from a large surface coal mine using a field spectroradiometer in laboratory. GEODERMA. 2017;288: 47–55. doi: 10.1016/j.geoderma.2016.10.033

[24] Wang HT. Predicting of forest soil organic carbon content based on Near Infrared Spectroscopy. Ha Erbing: Northeast Forestry University 2014 Available from: http://xueshu.baidu.com/s?wd=paperuri%3A%28a646797d4f863635b5bce693427a5b04%29&filter=sc_long_sign&tn=SE_xueshusource_2kduw22v&sc_vurl=http%3A%2F%2Fd.wanfangdata.com.cn%2FThesis%2FY2721781&ie=utf-8&sc_us=1298004871981196110.

[25] Yang AX, Ding JL. Comparative assessment of two methods for estimation of soil organic carbon content by Vis-NIR spectra in Xinjiang Ebinur Lake Wetland. Transactions of the Chinese Society of Agricultural Engineering. 2015; 31(18): 162–168.

[26] Chen HZ, Pan T, Chen JM. Combination optimization of multiple scatter correction and Savitzky-Golay smoothing modes applied to the near infrared spectroscopy analysis of soil organic matter. Computers and Applied Chemistry. 2011; 28(5): 518–522.

[27] Viscarra Rossel RA, Behrens T. Using data mining to model and interpret soil diffuse reflectance spectra. Geoderma. 2010; 158(1–2): 46–54. doi: 10.1016/j.geoderma.2009.12.025

